# Analysis of and Reduction in Noise in Current Measurement of XCP under the Laboratory Condition

**DOI:** 10.3390/s22228715

**Published:** 2022-11-11

**Authors:** Yunliang Liu, Guangyuan Chen, Libin Du, Qisheng Zhang, Keyu Zhou, Zucan Lin, Sheng Du

**Affiliations:** 1 School of Marine Science and Engineering, Shandong University of Science and Technology, Qingdao 266000, China; 2 School of Geophysics and Information Technology, China University of Geosciences (Beijing), Beijing 100083, China

**Keywords:** XCP, current measurement, electromagnetic interference testing, flume experiment design, noise analysis and correction

## Abstract

Expendable current profiler (XCP) is one of the most vital devices detecting ocean currents. Compared with other methods, the expendable feature makes trials with XCP much faster and more hidden, while the accuracy of XCP is considerably influenced by electromagnetic noise all around. Aiming at researching the influence and reducing the noise, this study carried out laboratory simulation experiments. The designed laboratory experiments mainly have a self-developed rotation gear, an XCP prototype, a plastic flume, and two copper plates as power. Firstly, these experiments analyzed the main sources of electromagnetic noise for XCP detection. Secondly, we built a noise simulation environment and conducted XCP detection experiments under different noise in the flume. The data obtained by XCP were transmitted to the computer to be stored and processed. The results show the internal noise impact on XCP is significantly less than the external. For an excitation power of 1 mV, the offset of theoretical and actual data brought by internal noise is 12 times smaller than external and can be corrected.

## 1. Introduction

**Figure 1:**
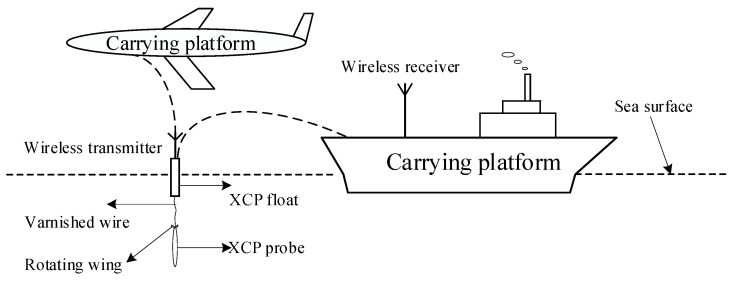
XCP is released and work diagram. Rotating wing: makes the probe rotate down. Varnished wire: transfers data. XCP float: wireless transmitter and probe release. Wireless transmitter and wireless receiver: wireless transmission and reception of data.

The expendable current profiler (XCP) is a disposable marine environmental measurement profiler that can quickly obtain sea current profile information. Because of its low cost, convenient operation, and real-time measurement, it is widely used in marine surveys and marine dynamic environment research [1,2,3]. As seawater moves massively in the geomagnetic field, it generates induced electric potential and induces currents by cutting the magnetic lines of force of the geomagnetic field. In the case of a stable geomagnetic field, the magnitude of the induced electric potential depends mainly on the velocity of the sea current. Therefore, the speed and direction of the ocean current can be calculated by measuring the size of the induced electric field generated by the ocean current [4,5,6], and the XCP is designed according to this principle. The sensor of the XCP probe is composed of two electrodes and a compass coaxial coil with an electrode. Its working mode is shown in Figure 1; it can be equipped on ships or aircraft for launching and releasing, because the probe tail is equipped with a rotating wing. As the XCP probe sinks in seawater, the rotating device at the tail of the probe drives the electrodes and compass coils to rotate and fall at the same frequency, cutting the geomagnetic field to produce a certain electrode signal and coil signal. Then, the data obtained from the XCP probe are sent through varnished wire and a wireless transmitter in the XCP float, and finally the data are received by the wireless receiver.

Thomas B. Sanford and others from the University of Washington in the United States proposed the basic calculation formula of the electromagnetic field induced by seawater movement in 1971 and conducted in-depth research on the principle and application of XCP [7]. At present, XCP products have been widely used in marine physics research in the Pacific, Atlantic, Arctic Ocean, and other seas [8,9]. However, the induced electric field signal generated by the ocean current cutting the geomagnetic field is very weak, and XCP is extremely susceptible to electromagnetic signal interference, which affects the measurement precision and accuracy. There are relatively few public studies on the numerical simulation analysis or laboratory simulation test of XCP noise interference effects in the world. The XCP performance test and application analysis are mainly applied to the seawater velocity profile observation in the oceans around the world [10,11]. The systematic research on XCP in China began in the early 21st century, and many universities and research institutes carried out related research and exploration [12,13,14]. National Ocean Technology carried out a numerical simulation study on the flow field of the XCP probe during the descending process and optimized the XCP structure design [15,16,17]. At the same time, by building an experimental device and applying high time resolution TRPIV (Time Resolution Particle Image Velocimetry) technology, the two-dimensional instantaneous flow field and average flow field of the horizontal section of the XCP probe were measured, and the convective flow field at different rotational speeds and Reynolds number was analyzed. This provides a basis for the falling posture [18]. The China University of Geosciences (Beijing) established an induced electric field model of current motion in XCP for weak ocean current signal extraction and proposed a more effective method for processing XCP data [19,20,21]. In addition, aiming at the influence of the high resistance of the XCP probe on the induced electric field in seawater, relevant research was carried out, and a reasonable probe size was designed according to the experimental results [22].

However, there are still relatively few laboratory flow velocity acquisition tests and laboratory flow velocity measurement experiments aimed at studying the influence of electromagnetic noise on XCP measurement. On the one hand, the reason is that the actual ocean current electric field signal is close to the nanovolt level, and it is difficult for the laboratory to provide a stable signal source. On the other hand, in the test environment in the onshore laboratory, it is impossible to filter out the high-frequency interference caused by the seawater, so there is more and more complex electromagnetic interference than the background electromagnetic noise of the ocean [23], which makes the extraction of weak current signals more difficult. However, as an intermediate environment linking theoretical simulation and ocean testing, the importance of building laboratory simulation experiments is self-evident.

Therefore, on the basis the preliminary theoretical research and the actual signal acquisition process of XCP, the XCP measurement method is simplified in this experiment. The designed laboratory experiments mainly have a self-developed rotation gear, an XCP prototype, and simulated electric field environment. By analyzing the possible electromagnetic interference of the XCP sensor in the marine environment, the noise source is simulated in the laboratory environment, and the measurement level and test performance of XCP under different electromagnetic interference are analyzed. The laboratory analysis and test method of XCP is further improved, which provides a new research idea for the effective extraction of ocean-current-induced signals under complex marine background noise.

## 2. XCP Measurement Principle

**Figure 2:**
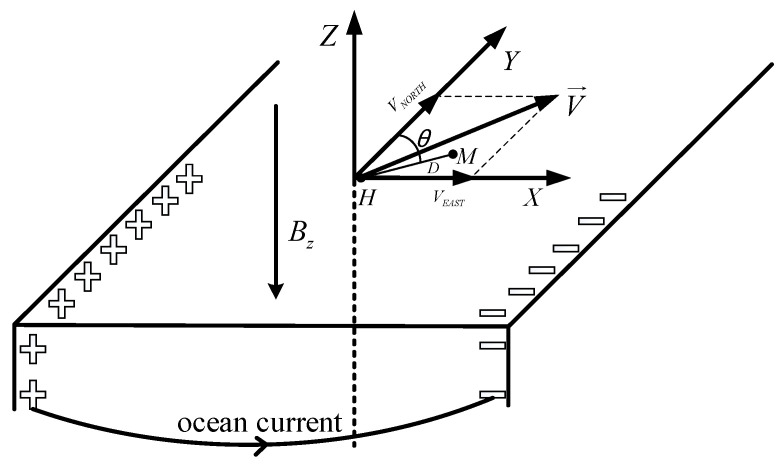
Currents cut by ocean currents. D represents the distance between the two electrodes H and M of the XCP electrode sensor, and θ represents an angle between the measurement direction of the induced voltage and the Y axis.

Combined with the working principle of the Hall effect, during the large-scale flow of seawater, the magnetic field lines of the geomagnetic field are cut to generate induced electromotive force and induced current. As shown in Figure 2, when the ocean current cuts the vertical component of the geomagnetic field, the accumulation of charges is generated on the horizontal sides of the ocean current, resulting in a horizontal-induced electromotive force, which forms a closed loop through the conductivity of seawater in different current layers and the seabed. Establishing a coordinate system as shown in Figure 2 involves the following: the X axis points east, the Y axis points north, and the Z axis points up. When the ocean current flows in the horizontal direction, the direction of the induced voltage and the Y axis generate an included angle (measurement azimuth θ); at this time, the ocean-induced electromotive force between the electrodes with a distance of D in the XCP sensor is shown in Formula (1) [24]:

(1) $$\begin{array}{l} \Delta \phi =\vec{D}•(\vec{V}\times \vec{B})\\ =B_{Z}DV_{EAST}\cos\theta -B_{Z}DV_{NORTH}\sin\theta \end{array}$$

Among these variables, $\vec{V}$ is the relative velocity at which seawater flows through the electrode, $V_{EAST}$ and $V_{NORTH}$ are the eastbound and northbound components of ocean current velocity $\vec{V}$, and $B_{Z}$ is the vertical component of the 3D geomagnetic field. In the mid-latitude region, the amount of induced electric field generated by 1 cm/s ocean current is less than 1 μV/m [25]. Since the current electric field sensor at the XCP probe end is about 5 cm, if the 1 cm/s ocean current needs to be distinguished, the measurement accuracy of the ocean current electric field sensor needs to be better than 50 nV. Therefore, the accuracy of the current signal is greatly affected by the electromagnetic signal, and it is very important to carry out the electromagnetic interference test research in the laboratory environment.

## 3. XCP Electromagnetic Noise Source Analysis

When the XCP is measured, the electromagnetic interference it receives mainly comes from the external and internal aspects of the XCP. The external interference mainly comes from the measuring ship. On the one hand, since the hull itself is a large metal body, in locally wet conditions and bilge water, steel comes into contact with carbon dioxide and the surface is surrounded by an acidic electrolytic solution. Because steel contains a small amount of carbon, when the surface is covered with a weakly acidic solution, a number of galvanic cells are formed on the surface. Carbon is positive and iron is negative, therefore, it loses electrons and becomes oxidized [26]. Therefore, redox reactions occur in the hull and corrosion currents are generated. On the other hand, because the ship is often equipped with high-power electrical equipment, it also interferes with ocean electromagnetic fields. The internal interference mainly comes from the flow velocity sensor mounted on the head of the XCP, and its sensing material and test circuit cause electromagnetic interference to the collection of weak sea-fluid induction signals.

### 3.1. Analysis of External Electromagnetic Field Source Interference

Due to the presence of more electrical equipment in the hull, it is equivalent to a larger source of external electromagnetic interference in the sea [27,28]. In this regard, when the XCP is launched, it has a great impact on the measurement of the offshore XCP, which seriously affects the measurement accuracy of the XCP sensor. With the continuous falling of the XCP sensor, the influence of external electromagnetic interference on the XCP sensor is also reduced due to the shielding effect of seawater and the distance of the XCP sensor from the hull. Therefore, it is of great significance to study the interference of the external electromagnetic field source on the measurement accuracy of the XCP sensor when the falling distance of the XCP is relatively short [29]. Therefore, in the laboratory environment, a DC motor is used to simulate the noise effect of the hull on the XCP sensor [30], and then the measurement accuracy and error data of the external electromagnetic interference in the simulated environment are analyzed.

### 3.2. Interference Analysis of Internal Electromagnetic Field Sources

In the signal acquisition system, the pre-conditioning circuit composed of operational amplifiers can improve the signal-to-noise ratio of the system and the transmission ability of the signal. The selection of operational amplifier directly determines the measurement accuracy and stability of the whole system [31]. Therefore, when the operational amplifier is used for XCP weak signal acquisition, the weak electric field signal can be amplified with a set gain, so as to reach the measurement range that can be extracted. However, the practical operational amplifier is not an ideal operational amplifier, and the input offset voltage error, common mode rejection ratio, and other related factors must be considered in the design.

**Figure 3:**
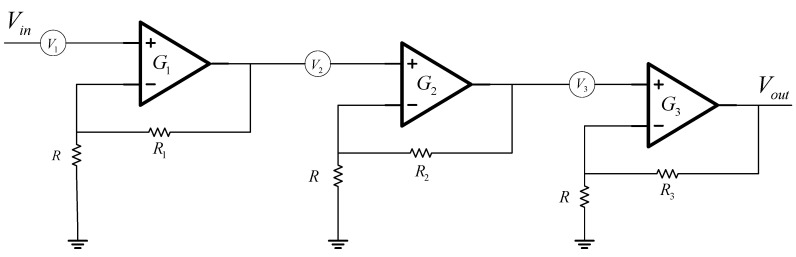
Simplified circuit of XCP op amp noise model: consists of a basic operational amplifier circuit whose magnification is mainly determined by two external resistors.

Since the actual current signal is extremely weak, it is generally at the nanovolt level. In order to collect this signal, it must be amplified enough, and the maximum gain of general-purpose operational amplifiers is generally less than 100 times. Therefore, when a higher gain is required, several operational amplifiers are generally cascaded to form a high-gain operational amplifier to meet the multiple gain requirements. Since the electrode signal amplifying circuit inside the XCP sensor is actually composed of a three-stage amplifying circuit, noise is inevitably introduced [32,33]. In order to study the noise effect introduced by the amplifier circuit, the noise effect can be converted into a noise voltage. That is, in a real circuit, the operational amplifier circuit can be equivalent to a circuit composed of a noise-free operational amplifier and a noise voltage source connected to the input terminal [34]. According to this theory, the noise model circuit diagram of the high-gain operational amplifier circuit is established, as shown in Figure 3 below:

In the figure above, $V_{1}$, $V_{2}$ and $V_{3}$ are the equivalent input noise voltages of the operational amplifiers at all levels, $G_{1}$, $G_{2}$ and $G_{3}$ are the closed-loop gains of the operational amplifier, $V_{in}$ is the input signal, and $V_{out}$ is the output signal. Assuming that $V_{N}$ is the output noise, then the total gain of the XCP internal circuit is $G=G_{1}\times G_{2}\times G_{3}$, and the output noise is

(2) $$V_{N}=V_{1}\times G_{1}\times G_{2}\times G_{3}+V_{2}\times G_{2}\times G_{3}+V_{3}\times G_{3}$$

Combining the above formula, we obtain $V_{N}=G(V_{1}+V_{2}/G_{1}+V_{3}/G_{1}/G_{2})$, it can be seen that the size of the introduced noise will increase with the increase in the operational amplifier, which affects the experimental results.

## 4. Noise Simulation Test and Result Analysis

### 4.1. Construction of Marine Noise Simulation Test Environment

According to the signal characteristics of the ocean current electric field signal and the compass coil signal, the electrode signal is the induced electric field signal generated by the ocean current cutting the geomagnetic field. The coil signal is generated by the compass coil cutting the geomagnetic field during the rotation and fall of the probe, and it can not only determine the direction of the current, but also collect the rotation frequency of the probe during its actual fall.

**Figure 4:**
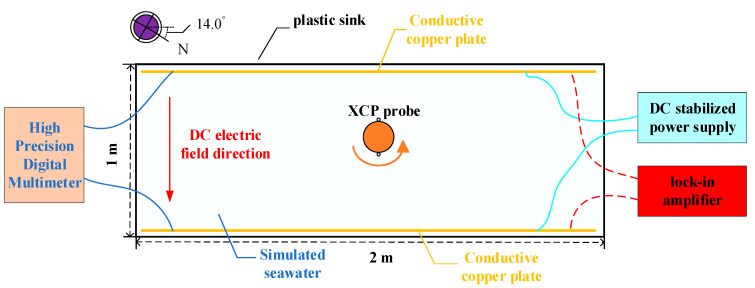
Schematic diagram of the construction of the marine simulation environment.

As the probe falls, it rotates and sinks at about 16 r/s; thus, the electric field signal and coil signal can be modulated into a narrow-band single frequency signal of 16 Hz, which reduces the noise of ocean current electric field signal and improve the signal-to-noise ratio. According to the measurement principle of the above XCP sensor, the construction of the laboratory water tank environment is divided into two parts. One part is the electric field environment composed of a non-metallic water tank with seawater, two conductive copper plates, a DC stabilized power supply, a lock-in amplifier, and a precision attenuator. The other part is a rotating device composed of a small DC motor, a motor speed controller, and a rotating bracket for an XCP probe. The schematic diagram of the marine electric field environment is shown in Figure 4.

The simulated seawater is salt water with a concentration of 3.5%, and the conductive copper plates are placed relatively in parallel. When the copper plates are powered by a DC regulated power supply, the two power supply copper plates pass through the seawater to form a stable DC electric field environment. When a lock-in amplifier is used to provide a stable AC signal for the copper plates, referring to the above, during the fall of the XCP probe, the probe rotates and sinks at about 16 r/s due to the tail rotating device. Thus, the electric field signal and coil signal are modulated into a narrow-band single frequency signal of 16 Hz. Therefore, the lock-in amplifier is used to provide a 16 Hz AC signal for the copper plate and coil. The DC electric field environment established in this experiment is based on the XCP measurement principle, and a stable DC electric field environment is constructed to replace the electric field environment generated by the ocean current motion cutting the geomagnetic field. The actual rotation acquisition of the induced electric field signal by the XCP sensor is replaced by constructing an AC electric field environment. On the one hand, it can verify the ability of the XCP electrode sensor to capture weak electric field signals, and on the other hand, it can better simulate the collection process of the XCP on the ocean current signal in the sea trial environment.

Since the XCP sensor is generally carried on the ship for sailing and discarding, during the sailing process of the ship, a large source of electromagnetic interference is formed due to the existence of generators and high-power electrical equipment. Therefore, the interference of the external electromagnetic field source may cause electromagnetic interference to the XCP sensor, affecting the current measurement results collected when close to the ship. To verify whether the ship in the sea trial environment causes electromagnetic interference, in the laboratory simulation environment, the DC motor is used to replace the external interference of the ship in the simulated environment. By analyzing the data collected by the XCP sensor under the interference of the DC motor, the influence of the external electromagnetic interference source on its measurement accuracy is obtained. At the same time, when the XCP sensor is far away from the ship and is not affected by its electromagnetic interference, since the marine environment is equivalent to a low-pass filter, it can better suppress the influence of external electromagnetic interference. Therefore, during the marine test of the XCP sensor, the noise introduced by its internal circuit design becomes the main source of noise interference. Therefore, if the DC motor that simulates the external interference source is removed, the internal noise test experiment and measurement result analysis can be carried out in the AC simulation environment.

### 4.2. Influence of Laboratory Background Noise

**Figure 5:**
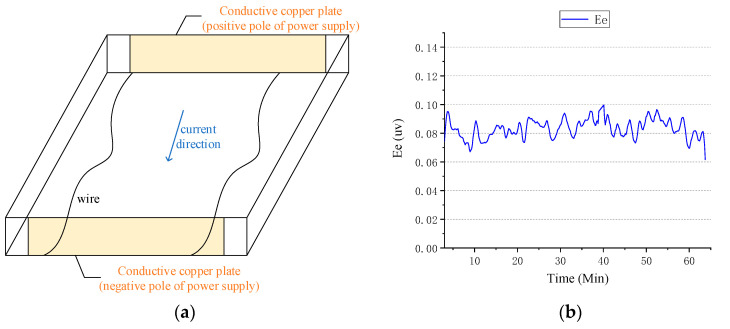
Laboratory background noise environment construction and test result analysis chart: (**a**) schematic of the background noise test. Conductive copper plate: construction of simulated ocean current electric field environment. Wire: connects two conductive copper plates. (**b**) Background noise test result plot: variation in background noise level over time in simulated environment.

Because in the offshore measurement of XCP, the seawater at a depth of one kilometer is enough to filter out most of the high-frequency noise interference, which is often impossible to achieve in laboratory simulations. Therefore, in order to carry out the research on the overall background electromagnetic noise interference in the laboratory environment, a test environment composed of non-metallic water tanks and prepared seawater was built. Two identical conductive copper plates were placed in parallel in the sink and connected by wires. Because of the existence of wires, a stable electric field environment can be established between copper plates in a short time. However, this did result in a significant drop in the voltage between the two conductive copper plates, and the electric field between the supply voltage of the copper plate and the actual copper plate had a certain deviation. Therefore, a high-precision digital multimeter was used to monitor the real voltage of the copper plates and obtain the actual electric field voltage value between conductive copper plates. In addition, in order to better simulate the conductivity of seawater in an experimental setting, tap water and salt were configured according to the salinity of seawater. By calculating the volume of tap water in a plastic tank, the weight of sea salt needed was determined based on an average salinity of 35% in seawater and the salinity in sea salt. After the copper plates were powered by a signal source, a stable electric field environment was formed between the two power supply copper plates, as shown in Figure 5a. Then, the XCP sensor was used to collect and analyze the data. The XCP sensor was used for continuous acquisition, and the data were organized to obtain the results shown in Figure 5b.

It can be seen from Figure 5b that after 60 min of data acquisition, the electrode voltage (Ee) between the induced electric fields measured by the XCP sensor in the simulated environment is stable between 0.06–0.1 μV. According to the calculation of the electric dipole distance, the noise level of the background electric field is about 1–2 μV/m. From Equation (1) and the values of the vertical and horizontal components of the Qingdao geomagnetic field, it can be inferred that the background electric field noise generated above has an impact of about 2 cm/s on the ocean current velocity. It shows that the terrestrial background noise still has obvious interference even for the water tank experiment, which increases the minimum threshold of the XCP flow velocity test experiment by 2 cm/s. The increase in the threshold value can be corrected by the processing of the measured data, thus an effective simulation of indoor flow rate measurement is realized.

### 4.3. Research on the Influence of External Electromagnetic Field Interference

**Figure 6:**
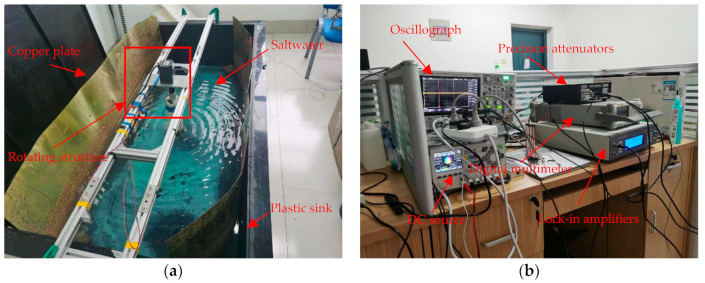
Laboratory simulation of ocean current environment construction diagram: (**a**) XCP simulated ocean current electric field and probe fixed rotating device. (**b**) XCP signal source and signal monitoring equipments.

As shown in the ocean simulation environment in Figure 6a, the laboratory simulation of the marine environment construction is mainly composed of the above four parts: copper plate, plastic sink, salt water, and rotating device. Among them, two copper plates placed relative to each other can be used to simulate the electric field of the sea current, and the rotating structure can drive the XCP probe to rotate at a stable frequency. In Figure 6b, the DC source, lock-in amplifier, precision attenuator, digital multimeter, and oscilloscope together constitute the signal source and monitoring equipment of the ocean current simulation environment. Among them, the DC source provides a signal source for the DC electric field experiment, the lock-in amplifier and precision attenuator provide the AC signal source for the AC electric field experiment, and the digital multimeter and oscilloscope can monitor the signal in real time.

In the acquisition process, since the DC motor drives the rotating structure for rotation acquisition, the external electromagnetic field introduced by the DC motor is used as the interference source, and the simulation analysis of the external electromagnetic field interference in XCP measurement is carried out. Since this paper mainly studies the influence of noise on the weak ocean-current-induced signal, the DC power supply voltage used to simulate the measured ocean current electric field signal is small, less than 1 mV. A high-precision digital multimeter was used to measure the actual voltage of the copper plate voltage and to compare and analyze the XCP acquisition signal with the theoretical value, as shown in Table 1.

**Figure 7:**
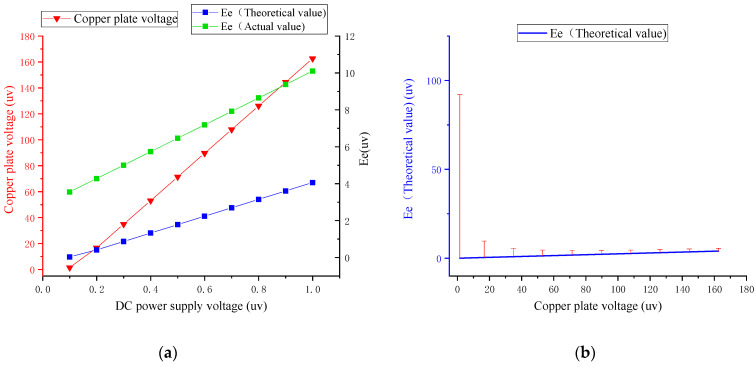
Analysis of DC electric field test results: (**a**) trend between copper voltage input and XCP-induced different voltage Ee; (**b**) error analysis of different XCP induction voltage Ee under copper plate voltage.

As shown in the above Table 1 and the following Figure 7a, with the reduction in the DC power supply voltage, the copper supply voltage is the actual external simulated electric field and is decreasing, and the electrode voltage collected by XCP also gradually decreases. When the voltage of the power supply copper plate is lower than 0.4 μV, the measured voltage does not change significantly with the reduction in the power supply voltage due to the influence of noise. In the process of the copper plate voltage decreasing from 1 mV to 0.4 mV, the measured induction signal decreases, and the relationship is approximately linear. There is a deviation of 6.0–4.4 μV between the theoretical induction value and the measured value, and the deviation value is relatively stable, with a variance of 0.344 μV. It can be eliminated by the error correction method.

By analyzing the relative error curve in Figure 7b, it can be seen that as the laboratory-simulated induced electric field strength decreases, the relative error caused by external electromagnetic interference gradually increases, and there is an obvious relative error turning point. Therefore, in view of the existence of external electromagnetic interference in the laboratory, based on the test results of the experimental numbers 1–7, the Allometric1 function was combined with the Levenberg–Marquardt optimization algorithm to establish the error model under the external electromagnetic field interference.

**Figure 8:**
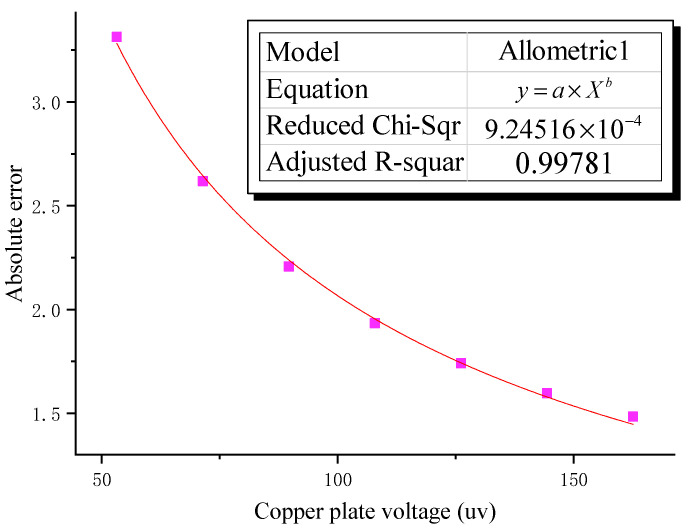
Error optimization fits the model result plot under external interference.

As shown in Figure 8, the relationship between the error caused by the external electromagnetic interference and the copper plate voltage is consistent with the Allometric1 model, and the adjusted R squared is closer to 1 by the Levenberg–Marquardt optimization algorithm. This shows that the error model fits well and is highly reliable. Therefore, on the basis of the above error fitting model, it can be obtained that in the process of laboratory and outdoor electromagnetic interference testing, the relationship with the voltage of the copper plate can be established by it, as shown in the following Formula (3), where δ is the error introduced under different copper plate voltages:

(3) $$\delta =(60.37186\pm 3.6)\;V_{\mathrm{Copperplate}}{}^{-0.73274}$$

### 4.4. Research on Influence of Internal Electromagnetic Field Interference

In order to study the interference of the XCP’s internal electromagnetic field on the measurement, a laboratory AC electric field environment was built for actual measurement. Different from the DC electric field environment, it does not require a rotating device to drive the XCP sensor for stable rotation acquisition testing. Instead, a lock-in amplifier and a precision attenuator are needed to provide the same frequency acquisition signal for the electrode signal and coil signal of the XCP sensor. Therefore, the influence of the main external electromagnetic interference is eliminated in the AC electric field environment. In order to better compare and analyze the experimental results of the DC electric field and the AC electric field, a lock-in amplifier was used to provide it with an input signal of the same frequency and voltage as the test frequency and voltage collected under the interference of the external field source, and real-time recording of the copper plate voltage with a high-precision digital multimeter was performed. By reading the data returned by the XCP probe and processing the data, the results are shown in Table 2 below.

**Figure 9:**
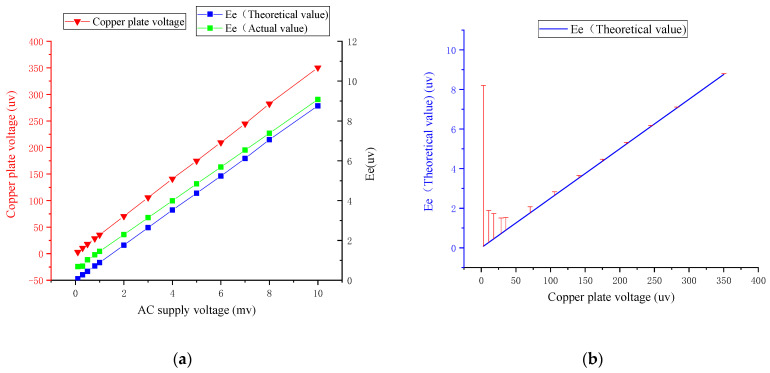
Analysis of AC electric field test results: (**a**) trend between copper voltage input and XCP induced different voltage Ee; (**b**) error analysis of different XCP induction voltage Ee under copper plate voltage.

As shown in the above Table 2 and the following Figure 9a, the AC electric field test results are obviously similar to the DC test results. AC input voltage, copper voltage, and electrode voltage measured by XCP sensor have the same downward trend. In a comparative analysis of real data and theoretical data from XCP, it was found that the intensity of the induced electric field measured by the XCP sensor is greater than the theoretical value, and the difference between the actual value and the theoretical value increases with the decrease in the copper plate voltage. Combined with the error analysis diagram shown in Figure 9b, when the absolute error is less than 1, taking the serial number 1–10 experimental data, the error model under internal electromagnetic field interference was established by the Allometric1 function combined with the Levenberg–Marquardt optimization algorithm.

**Figure 10:**
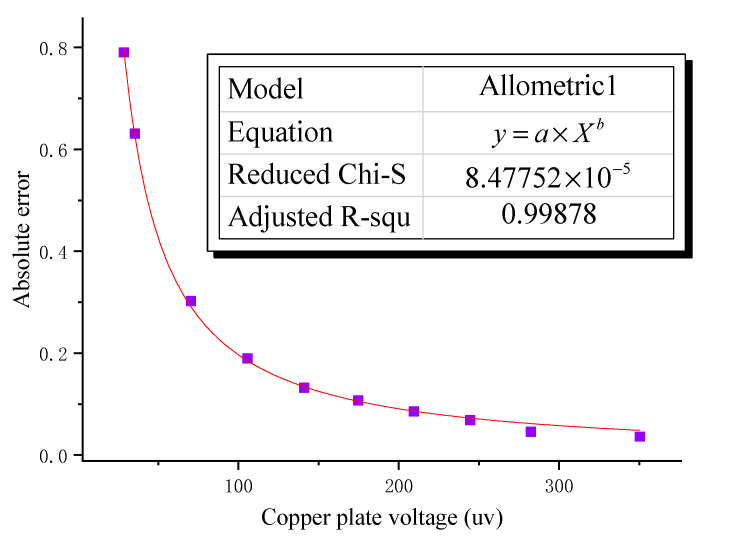
Error optimization fitting model result plot under internal interference.

As shown in Figure 10, there is a large correlation between the error caused by internal electromagnetic interference and the voltage of the copper plate. Therefore, combined with the above analysis of the error model under external electromagnetic interference, the relationship under internal electromagnetic interference is established, as shown in the following Formula (4), where δ is the error introduced under different copper plate voltages:

(4) $$\delta =(33.58408\pm 2.27161)\;V_{\mathrm{Copperplate}}{}^{-1.11606\;\pm \;0.01864}$$

Therefore, combined with the above error model, within a certain range of the copper plate voltage, the model can be used to correct the data of the XCP sensor laboratories without external electromagnetic field interference, so that the data processed by XCP can be within the allowable error range. The important thing is that because the same circuit boards are used in laboratories and outdoors, it can also guide the circuit optimization and data correction of the actual XCP ocean measurement to a certain extent.

## 5. Conclusions

In order to improve the measurement accuracy of XCP, this paper analyzes the interference factors of sea-fluid-induced electromagnetic field measurement. In view of the main interference factors, a laboratory physical test experimental environment was built, and the research on the influence of laboratory electromagnetic noise was carried out by using the developed XCP principle prototype. Through the signal acquisition test of the XCP sensor under different electromagnetic interference in the laboratory, the obtained data were processed, compared, and analyzed to achieve the following results:

1. The feasibility of the test method designed in this paper and the validity of the test system built in this paper are verified, and the influence analysis of electromagnetic noise in the XCP measurement process is realized.
2. The external electromagnetic interference source seriously affects the measurement accuracy of the XCP sensor by generating electromagnetic interference that is larger than the current electric field signal. For the applied electric field with an effective value of 1 mV, external noise interference causes a deviation of about 6 μV between the theoretical value and the measured value, and the measurement deviation has an approximate linear relationship with the measured signal. Therefore, by establishing an error correction model, the external electromagnetic interference noise is corrected, and the effective measurement of the laboratory flow velocity signal is realized.
3. Compared with the external electromagnetic interference, the noise error caused by the internal electromagnetic interference is smaller, the theoretical value and the measured value have a deviation of about 0.5 μV, and the measurement deviation has an approximate linear relationship with the measured signal. By establishing an error correction model, the internal electromagnetic interference can be effectively reduced, and the acquisition accuracy of the flow velocity signal can be improved.

In summary, both internal and external sources of electromagnetic interference have a non-negligible impact on the XCP measurement accuracy. Through laboratory measurement, a flow velocity error correction model can be established to reduce the influence of electromagnetic interference and improve the measurement accuracy of XCP. In the next step, it is urgent to carry out research on experimental simulation and data processing methods that can effectively shield electromagnetic interference, reduce or offset the electromagnetic interference on the road, and provide new research ideas for reducing the electromagnetic interference suffered by the actual XCP and improving the accuracy of ocean current measurement.

## Figures and Tables

**Table 1:** DC electric field test results.

Serial Number	DC Power Supply (mV)	Copper Plate Voltage (μV)	Ee (μV) Theoretical Value	Ee (μV) Actual Value
1	1	162.6165	4.0654	10.1004
2	0.9	144.3779	3.6094	9.3729
3	0.8	126.1393	3.1534	8.6453
4	0.7	107.9007	2.6975	7.9178
5	0.6	89.66216	2.2415	7.1902
6	0.5	71.42358	1.7855	6.4627
7	0.4	53.18499	1.3296	5.7351
8	0.3	34.9464	0.8736	5.0076
9	0.2	16.70782	0.4176	4.2800
10	0.1	1.53077	0.0382	3.5525

**Table 2:** AC electric field test results.

Serial Number	DC Power Supply (mV)	Copper Plate Voltage (μV)	Ee (μV) Theoretical Value	Ee (μV) Actual Value	Deviation
1	10	350.4321	8.7624	9.0756	0.3132
2	9	282.4158	7.0658	7.3804	0.3146
3	8	244.6265	6.1152	6.5328	0.4176
4	7	209.5485	5.2375	5.6853	0.4478
5	6	174.8124	4.3714	4.8377	0.4663
6	5	141.1245	3.5254	3.9901	0.4647
7	4	105.7285	2.6425	3.1425	0.5
8	3	70.5362	1.7625	2.2949	0.5324
9	2	35.5547	0.8875	1.4473	0.5598
10	1	28.5587	0.7137	1.2778	0.5641
11	0.5	17.9952	0.4497	1.0235	0.5738
12	0.3	10.8214	0.2725	0.704	0.4315
13	0.1	3.3214	0.0754	0.6845	0.6091
